# Collecting Feedback From Neurologists and Patients to Guide Development of a Parkinson Disease App (DigiPark): Qualitative, Noninterventional Study

**DOI:** 10.2196/55032

**Published:** 2024-12-31

**Authors:** Martin Duracinsky, Eva Brown Hajdukova, Fabienne Péretz, Julie Sauzin, Neziha Gouider-Khouja, Caroline Atlani, Djamchid Dalili

**Affiliations:** 1 Patient-Reported Outcomes Research, Health Economics Clinical Trial Unit, Hotel-Dieu Hospital, Assistance Publique – Hôpitaux de Paris, , Paris, France; 2 ECEVE, UMR-S 1123, Paris Cité University, Inserm, Paris, France; 3 MyPubli, Saint-Georges-sur-Baulche, France; 4 Abelia Science, Saint-Georges-sur-Baulche, France; 5 Care Lab, Paris Brain Institute, Paris, France; 6 Soukra Medical Center, Tunis, Tunisia; 7 Diampark, Paris, France

**Keywords:** DigiPark, Parkinson disease, patient-centered app, smartphones, usability testing, mHealth, mobile health

## Abstract

**Background:**

Parkinson disease (PD) is a worldwide, fast-growing, progressive neurodegenerative condition. Its multifaceted clinical presentation includes a wide range of motor and nonmotor symptoms. Smartphones present a potential solution to better monitor and subsequently alleviate PD symptoms.

**Objective:**

The aim of this study is to explore neurologists’ and patients’ needs and preferences regarding the design and functionality of a new smartphone app for PD, DigiPark.

**Methods:**

This qualitative, noninterventional study gathered data through two primary methods: (1) by conducting interviews with 9 neurologists and (2) through a usability test including 5 patients with PD.

**Results:**

The neurologists affirmed the necessity for a patient-centered app, highlighting the complexities of PD management. They advocated for personalized app functionalities to improve patients’ quality of life and emphasized the need for enhanced patient-provider communication. Feedback from the usability test indicated a preference for a clear, simple user interface, as well as elucidation of the app’s benefits. Concerns about the app’s time demands and the complexity of certain features like medication management were expressed. Furthermore, patients with PD consistently showed interest in features that could track and monitor their progress over time. This highlights the need to include clear benefits within the app to maintain user engagement and commitment.

**Conclusions:**

Neurologists’ and patients’ feedback on the design and functionality of the app complement each other. Collaborative efforts in shaping the app should better address genuine PD management needs. Future clinical trial inclusion can further validate the efficacy of DigiPark.

## Introduction

Parkinson disease (PD) is the fastest-growing neurodegenerative condition in the world [1]. According to 2019 global estimates, there were approximately 8.5 million patients with PD (PWPD) worldwide [2]. According to Dorsey et al, the global number of PWPD doubled from 1990 to 2015, and it is projected to more than double by 2040 [1]. The aging population, increasing longevity, decreasing smoking rate, and increased exposure to pollutants alone or in combination could all contribute to the huge increase in PD incidence [3].

PD is a heterogeneous and multifaceted disease, with rapidly and slowly progressive forms [4]. Clinically, the disease manifests through motor symptoms like tremors, slowed movements (bradykinesia), muscle stiffness, and difficulties with walking. Accompanying these are nonmotor symptoms, which range from sensory and neuropsychiatric abnormalities to sleep and autonomic disorders, including issues with bladder, bowel, and sexual functions [5]. In PWPD, symptoms and disability vary according to the stage of the disease, usually assessed by the Movement Disorder Society-sponsored revision of the Unified Parkinson Disease Rating Scale (MDS-UPDRS) [6,7]. Effective symptom management is imperative, as uncontrolled PD can lead to a decline in patients’ quality of life and heightened physical impairments [8]. However, managing both motor and nonmotor symptoms of PD is challenging and prescribed drugs induce noticeable side effects [9].

The variability of PD forms and symptoms, as well as drug-induced side effects, suggests opportunities for interventions to enhance patients’ quality of life [10]. Smartphone apps are widely used as clinical tools to better manage disease progression and optimize treatment. For PWPD, apps can foster collaboration, bridge communication gaps between patients and health care professionals, and track disease progression [11]. Using built-in sensors, like accelerometers and microphones, the apps can monitor PD symptoms seamlessly in daily life [12]. Using apps for continuous symptom detection and tracking addresses the sporadic nature of traditional clinical check-ups [13]. This is likely why a systematic review of apps tailored for PD available on Google Play and the App Store from 2011‐2016 found 125 apps; of these, 56 were potential aids for PD and 69 targeted PD directly, spanning information, assessment, and treatment categories [14].

The integration of digital health tools such as smartphone apps into the management of PD holds significant potential for enhancing patient care and improving communication between patients and health care providers. However, the usability of these apps for PD patients, particularly those with motor function impairments, is a critical factor that requires more comprehensive exploration.

Studies on the development process of apps generally lacked comprehensive methodological details, with a particular shortfall in the documentation of user feedback. The scientific evidence of their usefulness was also described as scarce and of poor quality, further highlighting the need to validate these tools and regulate their use [14]. Furthermore, to the best of our knowledge, there is no evidence in the literature showing the incorporation of user feedback from both neurologists and patients to guide the development of these apps.

DigiPark is a PD-tailored app that improves communication between health care professionals and PWPD. It has the potential to epitomize a paradigm shift in PD management, bridging diagnostic, therapeutic, and symptomatologic monitoring domains. The app acquired the Conformité Européene medical device status in October 2022. Following this acquisition, a systematic neurologist-patient collaborative feedback mechanism was initiated to ensure that the app evolved in tandem with user needs and expectations, thereby informing refinements for subsequent versions. The objective of this paper is to explore the impact of this feedback on the design and functionality of the app.

The objective of our study was to explore the needs and preferences of both neurologists and PWPD focusing on the design and functionality of the app, with the hypothesis that collected information would help enhance the clinical relevance and user-centricity of the app.

## Methods

### Study Design

This was a qualitative, noninterventional study collecting insights from neurologists and data from PWPD. These insights from neurologists were obtained from interviews and data from PWPD were obtained from patients’ usability testing.

### Ethical Considerations

This study does not correspond to any of the categories falling within the scope of research involving human subjects according to Article L1121-1 of the French Public Health Code and therefore no ethical approval was needed. Before participating, eligible participants provided their formal consent. Additionally, all procedures adhered to applicable privacy regulations and ethical standards. Participants' data were handled confidentially and stored securely, ensuring compliance with the General Data Protection Regulation and other relevant data protection laws. Participation was entirely voluntary, and participants had the right to withdraw at any time without any consequences. Participants did not receive any compensation for their participation in this study.

### Participants

All participants were identified and introduced to the study by a research institute specializing in neurological disorders (Paris Brain Institute). Only French-speaking individuals aged ≥18 years and neurologists practicing in France were included in the study, which ran from November 2021 to November 2022. All PWPD were to be naive regarding the use of the app (first-time users).

### Smartphone App

The DigiPark app platform incorporates educational resources, aiming to elucidate the complexities of PD and provide insights into individual symptomatology. An integral component, termed the “pillbox,” proffers real-time management of medication regimens, favoring timely adherence. Complementing this, the “symptom diary” enables daily symptom documentation, thus enabling a comprehensive longitudinal analysis of the patient’s disease trajectory. Further, a specialized feature, “tremor,” quantifies the magnitude and progression of a patient’s tremulous movements. Concurrently, the “phonation” module appraises vocal stability, while the “voice dictation” module critically evaluates pronunciation precision through structured exercises. The app is compatible with both Apple and Android smartphones, as well as Android smartwatches. It is a Conformité Européene–certified medical device.

The initial beta version of the DigiPark app (version 3.4) was orchestrated by Follow Product Management. This version provided a foundational understanding of user flow, particularly in the context of addressing medical necessities. The developmental team consisted of a data management expert, a chief technology officer, 3 developers (2 front-end and 1 back-end), and a user experience designer. During the different steps leading to this version, continuous feedback was paramount. Inputs were gathered from a cohort of beta testers, consisting of 10 PWPD, and an expert board meeting that included neurologists, PWPD, and app developers. Subsequent phases of development focused on the integration of key app features, namely, the pillbox, symptom journal, and rehabilitation exercise modules. To ensure the robustness and relevance of these features, feedback for version 3.4 was solicited through a survey from 16 neurologists affiliated with a French neurologist association (*Association des neurologues libéraux de langue française*).

### Usability Testing

A usability test (UT) to explore the usability of the DigiPark app (version 3.4) was conducted with PWPD. A list of essential tasks and features central to the app’s function was identified. These tasks represented key actions that users would likely engage with. A controlled environment for face-to-face interviewing was set up to eliminate potential distractions, ensuring participants could concentrate entirely on the app.

Before starting the UT, participants were briefed about its aim and given a general overview of the process, though they were not guided on how to use the app. It was highlighted that the goal was to evaluate the app, not the user’s proficiency. During the UT, participants interacted with the app and attempted to complete the predefined tasks. The observer took detailed notes on participants’ actions, challenges, successes, and any difficulties or points of confusion they encountered. After task completion, participants provided feedback on their experience. They were asked to describe any issues they encountered and suggest improvements.

Tests were to be done face-to-face or exceptionally conducted online. After each UT session, a summary was prepared. Only summaries were collected and archived. To maintain privacy, all collected data were stored and shared anonymously.

### Qualitative Interviews

Semistructured interviews were conducted with the neurologists in person or using online screen-sharing software. Interviews included 2 parts. During the first part of each interview, open-ended, exploratory questions allowed participants to discuss relevant issues in an open, unbiased, and spontaneous way. Explored topics included Parkinson diagnosis, treatment, and management. In the second part of the interview, the app’s medical content’s accuracy and pertinence were explored. Neurologists were also asked to provide suggestions for the app’s improvement. The interviews were audio recorded and subsequently transcribed verbatim.

### Analysis

Basic descriptive statistics were used to summarize the participants’ demographics. User feedback was aggregated, and common usability issues were identified, categorized, and prioritized based on their severity and potential impact on the user experience. Data from the neurologists collected during semistructured interviews were analyzed following a thematic analysis approach. Concepts were coded based on directed content analysis techniques to explore crude data [15]. A 5-step process was used: (1) analytic ideas and insights were identified in each transcript; (2) descriptive codes were allotted to quotes within transcripts; (3) the initial fit of the codes to the data was revisited throughout the coding process, and codes were merged or split into subsequent detailed codes; (4) concepts and domains relevant to the research questions were defined and refined using these codes; and (5) concepts and domains were reported alongside quotes, and counts were used where appropriate.

## Results

### Neurologists’ Interviews

In total, 9 neurologists (5 women and 4 men) were interviewed. All but 1 were practicing in the Paris area. Their years of experience ranged from 5 to 35 years (mean 16 years). Six practiced exclusively in a hospital, 2 split their time between a hospital and a private practice, and 1 worked in a private practice only. Five interviews were conducted in person and 4 were conducted using online screen-sharing software. Each interview lasted approximately 1 hour.

Themes, related information, and verbatim quotes from these interviews are detailed in Table 1 for the first and second parts of the interviews.

Insights from the first part of the interviews (Parkinson diagnosis, treatment, and management exploration) revealed 4 major themes: patient-centricity and communication; role of caregivers; clinical treatment and adherence; and diagnosis approach, therapeutic modalities, and follow-up procedures.

Patient-centricity and communication emphasized the importance of face-to-face communication to better understand patients’ day-to-day lives. It also revealed the importance of face-to-face interviews in understanding how the disease manifests in their patients and the importance of allowing patients to express their priorities and spontaneously report their complaints. Finally, the ethics of communication was another pivotal aspect of this first theme. Role of caregivers highlighted the significant contributions that caregivers can bring to consultations, especially when cognitive functions are compromised. One neurologist indicated that men were more likely to be accompanied by caregivers, which may lead to more comprehensive care, further highlighting potential gender disparities. Clinical treatment and adherence focused on medication adherence, which is a challenge due to possible cognitive function impairment. In addition, treating PD is complex and requires a patient-focused approach, effective communication, and personalized care. Finally, diagnosis approach, therapeutic modalities, and follow-up procedures grouped aspects related to the management of PD from diagnosis to monitoring (the disease’s cognitive, motor, and behavioral facets, plus a large range of existential concerns such as autonomy). Continuous care was also a common objective.

Insights from the second part of the interviews (feedback on the PD app) revealed 5 major themes: user-friendly design; data collection and presentation; integration into clinical practice; potential risks of overreliance; and additional features and functionality.

They showed that, globally, the app was perceived as a promising tool for managing PD. A consensus among neurologists was reached on the importance of user-friendly data presentation, the balance between objective and self-reported data, and the app’s utility in clinical practice. The diverse feature suggestions indicated the varied needs of both neurologists and PWPD. The app was commonly perceived as a supportive tool. In addition, the enduring importance of face-to-face consultations was noted, further highlighting that apps should only complement, and not replace, personal interactions between the doctor and the patient. The potential of the app in enhancing PD management was underscored, but it was commonly believed that, for its successful integration into regular medical practice, simplicity, relevance, and patient well-being should be prioritized while ensuring it complements the existing doctor-patient relationship.

Considering the diverse age range of PWPD and varying technological literacy levels, having a user-friendly design was perceived as paramount. A recurring theme across responses emphasized the necessity for the app to be simplistic (easy-to-understand, with a concise format). Concerns were raised about PWPD’s ability to use a digital tool due to the age of the PWPD population and the varying levels of technological literacy. The risk of noncompliance due to monotony was also evoked. In terms of data collection and presentation, neurologists expressed a preference for visualizing summarized data graphically. Although they were aware that the app was intended to serve as a data collection tool, they believed that ensuring data were presented in a meaningful and concise manner was critical. Four neurologists indicated the need for data to be directly applicable and beneficial during consultations. They commonly believed that, for an app like DigiPark to be integrated into clinical practice (ie, regular consultations), its data should not only be accessible but also be pertinent to treatment decisions. Furthermore, 2 neurologists expressed concerns about the potential risks of overreliance, which could lead to unnecessary focus or data misinterpretations. Finally, neurologists appreciated the app features and functionalities, including medication reminders, sleep data, joint exercises, and symptom tracking, but suggested a more integrative approach. Three neurologists expressed interest in a holistic app that not only tracks symptoms but also educates, offers rehabilitation exercises, and promotes engagement in therapeutic activities. One neurologist stated that the app should encompass a holistic approach to the patient’s overall well-being. They also believed that this could be the distinguishing feature that sets the app apart from other health management tools. The tool’s potential to monitor medication compliance was emphasized by several neurologists.

**Table 1.** Themes and related information enriched by verbatim quotes from the neurologists’ interviews.

Theme	Insights	Verbatim quotes
**PD^a^ diagnosis, treatment, and management exploration**		
**#**1. Patient-centricity and communication	Importance of face-to-face interaction: for a better understanding of patients’ day-to-day lives, for a better understanding of how the disease manifests in patients, and to allow patients to express their priorities and report complaints Ethics of communication: ethical aspect of explaining diagnoses thoroughly	I find that nothing beats an in-person interview. You must guide them, otherwise, things go in all directions. (004)I always take the time to explain... it seems unthinkable to me to give someone a diagnosis and then tell them ‘goodbye and see you in 3 months.’ (003)
#2. Role of caregivers	Importance of caregivers: caregivers are close relatives (family, friends); caregivers more frequently accompany men than women; caregivers are required in case of compromised cognitive functions	Men were more likely to be accompanied by caregivers, which may lead to more comprehensive care. (002)
#3. Clinical treatment and adherence	Challenges with medication adherence, as PWPD^b^ may have cognitive impairments and PD treatment is complex	One must always inquire about the patient’s actual intake because patients often don’t take the prescribed medications...Parkinson’s treatments can be complex. (009)
#4. Diagnosis approach, therapeutic modalities, and follow-up procedures	Different diagnostic strategies (eg, integrative approach and multiple clinical evaluations) Immediate hospitalization in case of severe symptoms Referral to an expert institution for specialized interventions Arrangement of appointments Issues in tracking symptom changes for patients Continuous care with biannual appointments and customized therapeutic strategies Monitoring of treatment compliance: differentiating between actual doses taken versus those prescribed, offering insights into patient adherence, facilitating the logging of medication timings with meals, and helping both the patient and health care provider fine-tune treatment plans	Understanding the specific mechanisms of a disease is secondary to the ability to alleviate the functional limitations it imposes. (003)
**Feedback on DigiPark app (version 3.4)**		
#1. User-friendly design	The app should have a simple design and concise data presentation. It should reduce the risk of monotony. It should consider the age of the population of PWPD and the varying levels of technological literacy.	In my opinion, it’s better to keep it [the app] simple because otherwise, people won’t use it if it’s too complicated. (007)For older people, it’s a little more complicated, and a Parkinson’s patient, even if they are young, are still behind you on many things, it is for that reason it [the app] must be simple.
#2. Data collection and presentation	Suggestions included visual presentation (graphics), concise presentation, synthetic data (averages), and color-coded indicators	There are [in the app] times for getting up and going to bed, but here I have no duration. Average sleep is displayed so what is missing is a bit precisely what we were saying a bit of synthesis. So, we need a way to synthesize this information in a somewhat graphic way. (002)...to have a day with a color code where in green is when the patient is okay and in red, it is when he is stuck, and let’s say in yellow he is experiencing dyskinesia, anything like that, but that could be it and then the app would do a bit of an average. (002)
#3. Integration into clinical practice	The app should be directly applicable during consultation, beneficial (support for treatment decision), and concise.	If the patient sends me more than two sheets, I will not read them. (006)
#4. Potential risks of overreliance	Risk of overreliance on the app, with potential psychological effects	...explicit feedback about the deterioration of a patient’s condition might adversely affect their psychological well-being. (008)
#5. Additional features and functionalities	Integrative approach: additional functionalities should be related to progression of symptoms relative to treatment timings, immediate evaluation of symptom severity (snapshot), education and engagement in therapeutic activities, and treatment adherence and monitoring	I think that an app for Parkinson’s must really integrate elements of a healthy lifestyle such as physical activity and giving exercise advice...to have little cardio exercises, meditation, things like that. (002)Perhaps we should have a little advice, that tells the patient to take their treatment before the meal. (002)

a PD: Parkinson disease.

b PWPD: Patients with Parkinson disease.

### Patients’ UT

Five PWPD (3 women and 2 men) completed the UT. All but 1 PWPD lived in the Paris area. They had had PD for 2-20 years. Four tests were conducted face-to-face and 1 was conducted online. Each session (ie, UT execution) lasted approximately 1 hour.

Insights from the UT provided a comprehensive understanding of the user experience. Findings were categorized into three main sections: (1) key findings, (2) observational insights, and (3) patient feedback and suggestions. This categorization helps to highlight the different aspects of the user experience, from interface design to functionality and user feedback. Each of the 3 sections was then split into several topics.

Considering key findings, several patients, especially those not digitally inclined, expressed discomfort with complex interfaces (user interface). The app’s purpose and benefit to the user was unclear to some patients (clarity of purpose), with certain sections necessitating a clearer explanation. Patients were apprehensive about the app’s demands, such as frequent logins, exercise routines, and detailed data input. They sought to understand the tangible benefits in return for their commitment (commitment concerns). A prominent expectation was a prefilled pillbox feature where patients could input their prescriptions, receive dose reminders, and confirm their intake (medication management). However, nuances such as understanding dosages presented challenges. There was a unanimous lack of interest in recording wake-up and sleep times. To entice users to consistently provide this information, a clear benefit must be demonstrated (routine inputs). A common desire was the ability to view longitudinal data, showcasing their improvement or decline (progress monitoring). This feature was deemed crucial to motivate continued engagement, especially for tasks like tremor and phonation measurements. Though seen as an enjoyable task, patients sought enhancements of the dictation function (voice dictation). Suggestions included gamification and variable difficulty levels. However, the significance of certain functionalities, like voice dictation, was lost on some, especially those without speech issues. The last topic highlighted the need for expanded exercises. In addition to specialist exercises, participants expressed an interest in physiotherapy, calligraphy, voice exercises, and more. Two patients specifically proposed exercises like calligraphy and voice modulation.

Considering observational insights, the first identified topic was general usability. Apprehensions about the app’s time demands were evident. For instance, some participants struggled with installation and many were hesitant in their interactions, possibly due to a fear of making errors. The necessity of improving homepage navigation was also pointed out. Identifiable clickable areas posed issues. For example, 1 participant clicked on text instead of the designated icon. Regarding the pillbox feature, while the process of adding levodopa was straightforward for some, challenges arose in specifying doses. Several patients found modifying medications challenging, describing it as “technical.” Certain functionalities, like the “restart” button postmeasurement, were misinterpreted, leading to patient distress. Moreover, the medical jargon used was confusing for many. Finally, for feedback and interpretation, patients were left perplexed by the numerical results in sections such as tremor and speaking; they sought qualitative descriptors or benchmarks for a better grasp of the implications.

Considering patient feedback and suggestions, PWPD faced display glitches, with 3 of them specifically emphasizing the importance of pill intake time customization. Although 4 patients emphasized the need for pill reminders, most were against reminders for measurements, fearing added pressure (functional challenges). The voice dictation exercise intrigued many, but its execution left room for improvement. Only 1 patient found the exercise game-like, while others sought more engagement (exercise feedback). Finally, proposals included calligraphy (suggested by 1 patient) due to its relevance to micrographia, sentence repetition, physiotherapy exercises, and more (patient-requested additions). Importantly, multiple patients welcomed the idea of a PDF report for neurologists. One patient, not well-versed with digital platforms, wished for paper-based exercises. Other unique suggestions encompassed a breathing test and voice amplitude/modulation exercises.

Table 2 presents examples of verbatim quotes issued from the UT of the DigiPark app (version 3.4) by section and topic.

**Table 2.** Examples of verbatim quotes collected by section and topic during patients with Parkinson disease utility testing.

Topic	Insights	Verbatim quotes
**Key findings**		
#1. User interface	Need for a clear, simple, and bug-free user interface	The app interface is too complicated for me to use comfortably.
#2. Clarity of purpose	Need for clearer explanations (benefit for the user)	I don’t see the benefit of logging in so often if it’s not clear how it will help me.
#4. Medication management	Patients requested a prefilled pillbox feature but faced challenges with understanding dosages	It’s hard to know if I’m inputting the correct dosage information.
#5. Routine inputs	Little interest in recording wake-up and sleep times unless benefits are demonstrated	I don’t want to record these every day unless it really helps.
#6. Progress monitoring	Viewing longitudinal data is crucial for continued engagement	Seeing my progress over time would motivate me to keep using the app.
#7. Voice dictation	Enjoyed by patients, but enhancements are needed, such as gamification	It would be more engaging if the voice exercises were more like a game.
**Observational insights**		
#1. General usability	Concerns about the app’s time demands and difficulties with installation and navigation	It took me a while to figure out how to even start using the app.
**Patient feedback and suggestions**		
#1. Functional challenges	Patients suggested additional features like calligraphy exercises, sentence repetition, physiotherapy, and a PDF report for neurologists.	I’d love to have a report I can print out and show my doctor.

## Discussion

### Overview

The recent drive toward patient-centered care has established the need for a well-structured approach in the management of chronic diseases such as PD. This study explored the neurologists’ and patients’ needs and preferences regarding the design and functionality of a smartphone PD app, DigiPark. These insights were then used to guide the development of the PD app. Our findings highlighted that both neurologists and PWPD believe in the potential benefits of the app; however, there are specific areas of refinement that we identified to ensure its practical usability and alignment with individualized, patient-centered care.

### Principal Results

Neurologists’ insights provide a clear road map for the app’s future evolution. Their emphasis on user-friendly design, data presentation, and synthesis, as well as the app’s integration into clinical practice, underscores the importance of translating raw data into actionable insights. Given the prevalent nature of PD among older adults [16], the expressed concerns about the technological literacy of this demographic reiterates the importance of simplicity in design and functionality. These sentiments are consistent with the literature on medical app usability, where user-centric design tailored to the targeted demographic’s needs is fundamental for effective adoption [17]. Contemporary recommendations for the development and rollout of digital technology emphasize the importance of collaborative design methods to create impactful health apps; focusing on user-driven design can enhance the usability of an app, making it intuitive, comprehensible, efficient, and well-received [18].

Another noteworthy point is the balance between self-reported data and objective metrics. The neurologists’ insights emphasized the balance between objective and self-reported data and usability; they also identified the need for the data to be synthesized in a way that is meaningful. Although digital tools provide an unparalleled opportunity to capture empirical data, the subjective experience of the patient remains vital. Therefore, a patient’s self-reported experiences should remain at the core of personalized care while also being harmoniously integrated with technology-driven objective data [19]. The value of personalized feedback and the potential risks of misinterpretation of data were also identified by neurologists, a sentiment that has been echoed in previous literature concerning health apps [20,21].

Furthermore, the contrasting feedback from neurologists and patients provides a comprehensive perspective. Neurologists focused on the clinical relevance of data, underscoring the app’s potential as a supplementary tool to enhance patient-doctor communication. In contrast, patients emphasized usability, clarity of purpose, and tangible benefits for their commitment. It is essential to recognize and address these distinct viewpoints to ensure the DigiPark app will be deemed both clinically valuable and user-friendly. The feedback also illustrates a universal need for data to be presented in a manner that is easy to interpret, both for patients and neurologists. This resonates with the assertion that the design of health apps should be intuitive [22], minimizing the cognitive load for users [23].

Patients’ concerns regarding the app’s commitment demands, as well as their unanimous lack of interest in recording certain routine inputs, signaled the importance of perceived value in driving consistent engagement. A similar observation was noted by Simons et al, who found that patients often prioritize functionalities that offer direct, immediate benefits, particularly when using digital health tools [20].

Clarity of purpose and clear benefits of the app usage were emphasized. Medication management was another area of concern, echoing the sentiment of the importance of clear medication guidelines for patients with chronic diseases [24]. Although the potential of the app in optimizing disease management was evident, study findings underscored the enduring significance of personal doctor-patient interactions. This echoes Street et al’s assertion that technological interventions should be seen as an adjunct to care and not a replacement, preserving the irreplaceable human touch in health care [25].

The demand for a prefilled pillbox feature highlighted the pressing need for medication management tools within the app. This aligns with the neurologists’ perspective, where monitoring medication compliance was emphasized. However, the challenges faced by patients in understanding dosages underscored the importance of developing an intuitive and straightforward medication logging system. Such considerations can enhance treatment adherence, where effective digital reminders significantly improve medication compliance among patients [26].

Patients’ desire for a longitudinal view of their progress and feedback aligns with the motivation theory in health behavior, suggesting that showcasing improvement or highlighting areas of concern can catalyze consistent engagement [27]. Furthermore, the diverse range of exercise suggestions (calligraphy, voice exercises, etc) indicates that PWPD are seeking holistic approaches beyond traditional therapeutic interventions.

### Study Strengths and Limitations

The insights gathered offer a substantial basis for the development of the DigiPark app, but it is essential to recognize several limitations that can influence the interpretation and application of these findings. First, our sample size was on the smaller side, in particular for PWPD. The decision to choose a sample size of 5 was informed by Nielsen’s usability research, which suggests that a group of just 5 users can reveal approximately 85% of usability issues. Nielsen’s research also points out that larger sample sizes often yield repetitive feedback with diminishing returns [28]. Hence, the choice of 9 neurologists and 5 PWPD for our study was strategic, aimed at obtaining an in-depth understanding of the app’s usability while ensuring resource efficiency. However, this means that our results might not effectively encapsulate the perspectives of all neurologists or the diverse experiences and needs of the broader PWPD community. This is particularly true for PWPD because the usability of the app may be impacted by motor symptoms such as rigidity or tremors directly (eg, inability to interact with touch screens) or indirectly due to frustration and reduced engagement with the app [29,30]. In this context, the DigiPark app must incorporate design features that account for the varying degrees of motor impairment in PD patients. This could include voice-controlled features or the use of external devices that ease interaction with the app for those with significant motor challenges. Furthermore, continuous UT with a diverse group of PWPD, reflecting different stages of the disease as per MDS-UPDRS classification, is essential to refine the app’s functionality and ensure it is accessible to all potential users. It should be noted that in the steps that followed the evaluation of the version used in this study, the app was continuously improved (more than 700 users by 2023). The PWPD who used the app reported about 800 suggestions or issues for improvement that were almost all resolved. The suggestions and issues included different categories such as improvement of symptoms presentation; improvement and new functions of the app; clarifications of vocabulary; better design; presenting of scores of gaming functions; user notifications; improvement of the patient interface; and improvements suggested by their treating clinicians. The severity of these patients was not directly measured; however, we inferred the disease severity based on the total daily dosage of levodopa administered. Our results showed that 75% of patients were in the early stage of the disease (Hoehn and Yahr score <2.5), while 10% were at an advanced stage (Hoehn and Yahr score >3.5), indicating that patients with more advanced disease stages could use the app [31]. Further compounding this limitation is the study’s predominant focus on the Paris region. Although valuable, this geographic concentration may not be fully indicative of the preferences, requirements, or challenges faced by users on a global scale. Methodologically, it is important to note that, while UT provides critical data on usability, it does not necessarily paint a comprehensive picture of the overall user experience. However, the UT method chosen for this study allowed for direct observation of how real novice users interact with the app. This method also provided valuable insights into the user’s needs, preferences, and challenges, which are crucial for improving the design and functionality of the app. The International Organization for Standardization describes usability in terms of efficiency, effectiveness, and satisfaction [32]. Early usability evaluations can pinpoint interface design issues throughout the development cycle [33] and are essential for user acceptance of health information systems and their safe, effective usage [34]. The feedback from the UT was used to make necessary adjustments and enhancements to the app, thus ensuring that it is user-friendly and meets the needs of the target user group.

### Implications and Perspectives

Although one of the main functions of the app, tremor evaluation, is measured by the watch (the patients do not need to fill in many fields in the app, but rather just need to click a few times), and various features have been designed to account for different clinical impairments, these results have led to the incorporatation of design features that account for the varying degrees of motor impairment in PWPD. The need for typing was reduced to a minimum, allowing patients to select most responses from predefined lists. To accommodate tremors, the interface was simplified, buttons were made larger, and navigation between screens was made more straightforward. Our results also led to the simplification of wording, the use of simpler vocabulary, and adjustments to syntax. For the adherence diary, commercial drug names will appear after typing just a few letters, with suggestions presented in a way that is easily recognizable. Similarly, to confirm medication intake, a simple click will suffice. The presentation of speech therapy exercises was also simplified for ease of use. Nevertheless, continuous UT with a diverse group of PWPD, reflecting different stages of the disease as per MDS-UPDRS classification, is essential to refine the app’s functionality and ensure it is accessible to all potential users.

Additionally, the digital health landscape is constantly and rapidly evolving, in particular in the context of chat-style interactions with large language models. As technologies advance and become more integrated into everyday life, expectations of users (here, PWPD and neurologists) also change. This dynamic environment means that these apps must remain adaptable, prepared for frequent updates, improvements, and major revisions driven by new insights, advancing technologies, and changing user needs.

Finally, our results have obtained new perspectives not only for the DigiPark and other smartphones apps but also for other neurodegenerative diseases. Throughout its history, PD has been a model for the development of advances in physiopathology, pathogeny, and therapeutics for other neurodegenerative diseases [35,36]. With a lesser impact, but similarly, the development of digital tools for PD, which aim to master and include as many as possible aspects of a very complex disease with a holistic and patient-centered approach, gave us several ideas and inspirations for the development of similar apps for other neurodegenerative or clinically heterogeneous diseases.

### Conclusions

Our study showed that the collection of feedback from both PWPD and neurologists during the development of a new PD app can bring rich and valuable information for the further development and refinement of a PD app (ie, DigiPark). Although the DigiPark app aims to bridge the gap between patient necessities and clinical PD management, it is imperative to conduct rigorous testing within a clinical trial setting. This is essential not only to validate its effectiveness and safety but also to establish its place in standardized care protocols. By including the app in a clinical trial, objective data on its impact can be generated and any areas for improvement can be identified. This will ensure that the app is not only effective but also held to the highest medical and technological standards, ultimately benefiting both patients and health care providers.

## Abbreviations

MDS-UPDRS: Movement Disorder Society-sponsored revision of the Unified Parkinson's Disease Rating Scale
PD: Parkinson disease
PDPW: patient with Parkinson disease
UT: usability test
